# Energy‐based corridor identification for mammals between protected areas in Iran

**DOI:** 10.1002/ece3.11551

**Published:** 2024-06-10

**Authors:** Ehsan Rahimi, Pinliang Dong, Faraham Ahmadzadeh

**Affiliations:** ^1^ Environmental Sciences Research Institute Shahid Beheshti University Tehran Iran; ^2^ Department of Geography and the Environment University of North Texas Denton Texas USA

**Keywords:** barriers, corridor mapping, energy usage, linkage mapper, pinch‐points, protected areas

## Abstract

Body mass plays a crucial role in determining the mass‐specific energy expenditure during terrestrial locomotion across diverse animal taxa, affecting locomotion patterns. The energy landscape concept offers a framework to explore the relationship between landscape characteristics and energy expenditure, enhancing our understanding of animal movement. Although the energy landscape approach solely considers the topographic obstacles faced by animals, its suitability compared to previous methods for constructing resistance maps and delineating corridors has not been comprehensively examined. In this study, we utilized the enerscape R package to generate resistance maps in kilocalories (kcal) by incorporating digital elevation models (DEMs) and body size data (kg). We assigned body sizes ranging from 0.5 to 100 kg to encompass a wide range of small and large mammals in Iran, adjusting maximum dispersal distances accordingly from 50 to 200 km. By analyzing these scenarios, we produced four resistance maps for each body size. Next, we identified potential corridors between terrestrial protected areas in Iran using the Linkage Mapper toolkit and examined barriers and pinch‐points along these paths. Our study revealed significant findings regarding the shared corridors between small and large mammals in Iran's landscape. Despite their differing body sizes and energy requirements, many corridors were found to be utilized by both small and large mammal species. For example, we identified 206 corridors for mammals weighing 500 g, which were also recognized as the least‐cost paths for 100 kg mammals. Thus, embracing a comprehensive method in resistance map creation, one that incorporates species‐specific traits and human infrastructure becomes imperative for accurately identifying least‐cost paths and consequently pinpointing pinch points and barriers.

## Graphical abstract

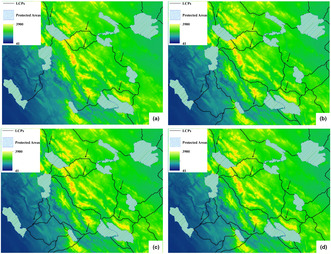

## 1 INTRODUCTION

Recent research conducted in Iran indicates that various human infrastructures such as roads, railways, urban areas, industries, mines, and agricultural activities have notable impacts on the country's protected areas (Karimi & Jones, 2020; Rahimi et al., 2023; Rahimi & Dong, 2022). These studies emphasize that such human activities can affect approximately 5.1%–30.3% of the total extent of protected areas in Iran (Rahimi & Dong, 2022). Karimi and Jones (2020) also demonstrated that human activities leave a footprint on 12.8% of national park areas, 12% of wildlife refuge areas, and 28% of the overall protected area extents in Iran. The consensus is that effective protection of species, ecosystems, and habitats can only be achieved when protected areas are functionally connected (Resasco, 2019). Therefore, it is advisable to implement practical measures aimed at preserving, improving, and restoring spatial networks between protected areas (Tabor, 2018).

Numerous investigations have tackled the task of identifying corridors and employing least‐cost modeling techniques for mammal populations. Typically, the process of mapping corridors involves two primary inputs: a resistance map and core habitat areas (Cushman et al., 2013). To generate a resistance map, some studies assess the different land use and land cover classes based on their potential to impede animal movement (Ersoy et al., 2019; Liu et al., 2018). Conversely, other studies utilize species distribution modeling (SDM) (Zurell et al., 2020) to develop a map of habitat suitability, which is then inverted and utilized as a resistance map (Mahmoodi et al., 2023; Rahimi & Dong, 2023). Typically, a separate resistance map is generated for each species, and habitat connectivity modeling for these species is conducted independently. When it's impractical to delineate corridors for all species, the focal species approach is commonly employed, where a subset of species is selected as an umbrella or indicator species (Khosravi et al., 2018). For example, Hosseini et al. (2019) created habitat suitability maps utilizing SDMs for four Persian leopard species, goitered gazelle, bezoar goat, and urial, and then applied circuit theory to pinpoint potential corridors linking protected areas. Khosravi et al. (2018) developed suitability maps using Species Distribution Models (SDMs) for leopard, cheetah, caracal, wild cat, sand cat, and wolf species in central Iran.

How animals navigate and interact with their environment plays a crucial role in shaping their impact on ecosystems, making it a fundamental aspect of biodiversity (Schlägel et al., 2020). The expenses associated with movement frequently rely on the environment that an animal traverses (Gallagher et al., 2017). Body mass plays a significant role in determining the mass‐specific energy expenditure associated with terrestrial locomotion across diverse animal taxa, which exhibit notable variations in leg structure, skeletal composition, and body temperature (Berti et al., 2022; Santini et al., 2013). On flat terrain, larger animals experience lower mass‐specific locomotion costs due to enhanced muscle efficiency as a result of longer stride lengths, enabling them to achieve comparable running speeds with reduced stride frequencies. This leads to longer periods of foot contact and more efficient muscle force generation (Halsey, 2016). However, when traversing uneven surfaces, larger animals expend additional energy due to the amplified effects of gravity (Snyder & Carello, 2008).

A proposed framework for studying animal movement, known as the energy landscape concept, aims to explore the connection between the physical characteristics of landscapes and the energy expenditure associated with animal movement, thereby enhancing our comprehension of their locomotion patterns (Gallagher et al., 2017; Shepard et al., 2013). The energy landscape provides a quantitative measure of the energy expenditure involved in movement within a specific spatial habitat, which is determined by examining how metabolic and biomechanical processes are influenced by various physical environmental factors (Berti et al., 2022; Gallagher et al., 2017). The energy landscape concept extends these predictions to geographic areas, incorporating topographic features to define a cost surface that shapes animal movement (Wall et al., 2006). This cost surface influences animal behavior, such as their avoidance of energetically demanding paths.

Recently, Berti et al. (2022) introduced a new R package called enerscape, which offers a framework integrating locomotory theory with Geographic Information Systems (GIS) to create energy landscapes tailored for terrestrial animals within specific spatial contexts. This integration involves combining an established model for estimating energy costs of movement (Pontzer, 2016) with a transition graphs approach and utilizing GIS tools available in R. The enerscape package enables the derivation of energy landscapes by utilizing elevation data and the body sizes of animals. Notably, the model for calculating energy costs of movement is incorporated as a module within enerscape, making it versatile and not restricted to terrestrial animals. This flexibility allows for potential expansion to include other ecosystem types and modes of movement.

While the energy landscape approach only accounts for topographic challenges encountered by animals, the applicability of this approach to previous methods for creating resistance maps and identifying corridors has not been thoroughly investigated. Since this approach only considers energy and topography to create resistance maps, there may be limitations as it overlooks human infrastructure such as roads that can impede animal movement. For instance, it remains unclear whether small mammals and large mammals would select different least‐cost paths based on this approach. Our hypothesis suggests that there would be no distinction between least‐cost paths for small and large mammals, as this approach minimizes resistance to the lowest elevation for any topographic map, regardless of the energy expenditure required by mammals. Consequently, corridor mapping software like Linkage Mapper (McRae & Kavanagh, 2011) would likely identify the same paths for both small and large mammals. If this holds, then barriers and pinch points along these paths would also be identical for both types of mammals. However, this approach is relatively new and has only recently been introduced in the enerscape R package. There is currently no comprehensive study utilizing enerscape as a resistance map for mammals. This study addresses this significant gap and has the potential to provide new insights into landscape connectivity modeling.

## 2 METHODS

### 2.1 Study area

**FIGURE 1:**
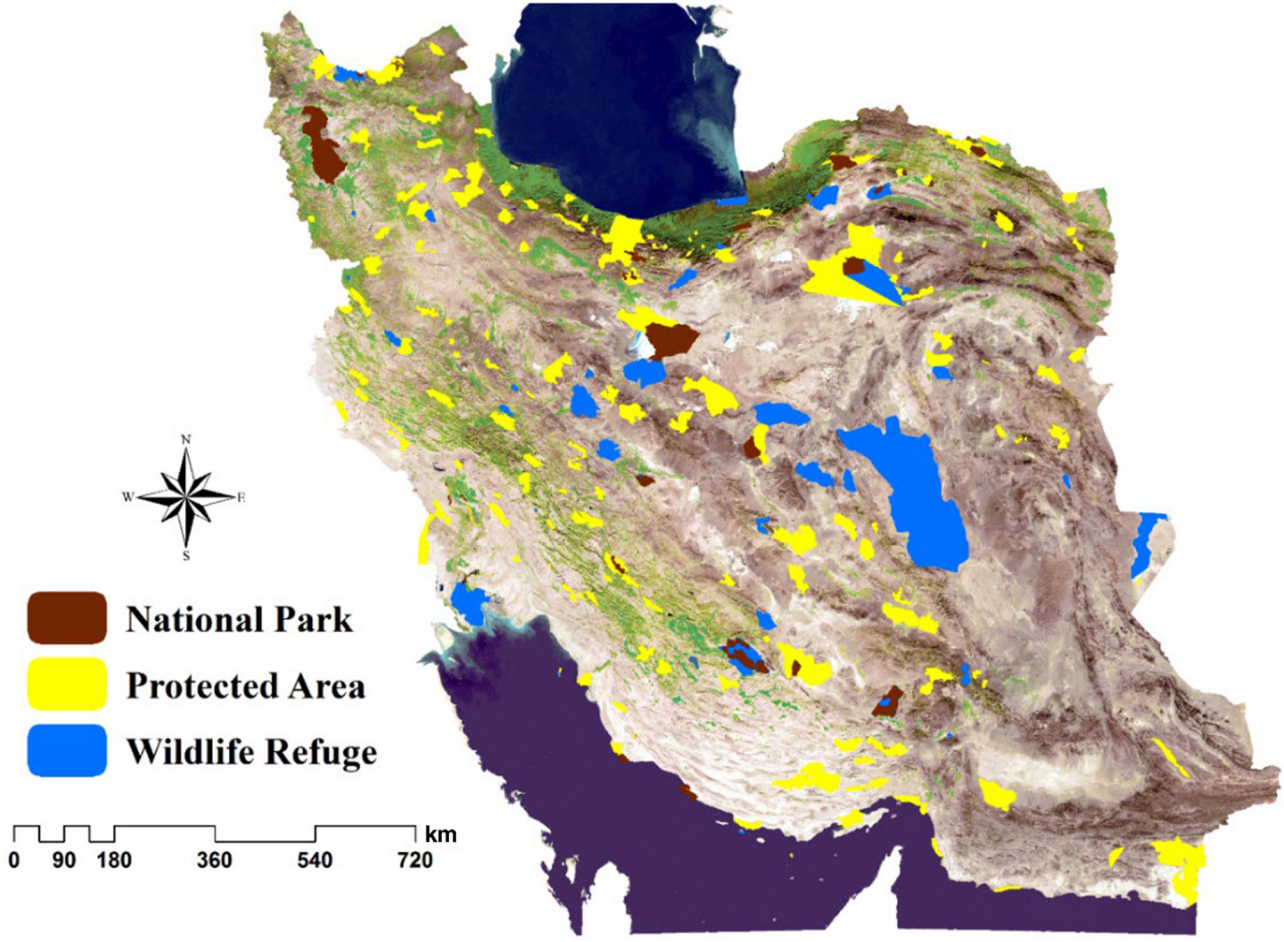
The location of national parks, wildlife refuges, and protected areas of Iran.

Iran serves as a vital bridge connecting the oriental areas to the African zoogeographical regions (Mittermeier et al., 2011). Iran boasts 169 protected areas, including 46 wildlife refuges and 31 national parks, covering a total area of 177,137.7 km^2^ (Iran's Department of Environment, https://doe.ir). Iran boasts an array of biodiversity, encompassing approximately 8000 plant species, 535 bird, 197 mammal, 227 reptile, and 21 amphibian species within its diverse ecosystems (Farashi & Shariati, 2017). The mammalian fauna of Iran comprises 192 species from 90 genera, 34 families, and seven orders, with 13% classified as endangered (Yusefi et al., 2019). Figure 1 illustrates the distribution of protected areas in Iran, showcasing the spatial separation of national parks, wildlife refuges, and protected areas.

### 2.2 Landscape connectivity modeling

Spatial networks are comprised of core habitats and corridors (Hilty et al., 2020; Sütünç, 2021). In this study, core habitats are all terrestrial protected areas of Iran, including national parks, wildlife refuges, and protected areas. Wetlands and rivers were not considered because they contain more water bodies than terrestrial habitats for mammals. To determine the corridors between protected areas, various methods such as least‐cost modeling, graph theory, and circuit theory are used. In this study, we use the least‐cost method to identify corridors, which is the dominant method in corridor modeling (Cushman et al., 2013). By accounting for landscape features and species movement preferences, this method offers a more ecologically realistic representation of corridors (Kwon et al., 2021). The least‐cost method requires a resistance map that provides a raster representation of landscape features in which the value of each cell indicates an estimate of the cost of species moving through that cell.

### 2.3 Resistance map

For the creation of resistance maps, we utilized the enerscape R package (Berti et al., 2022), which requires readily available topographic and body size data. Initially, we downloaded the Digital Elevation Model (DEM) of Iran using the geodata R package (Hijmans et al., 2015), with a cell size of approximately 900 m with GWS184 CRS. Subsequently, we considered four scenarios for creating resistance maps. The enerscape R package takes into account the DEM and body size data (kg) to generate resistance maps in kilocalories (kcal) or joules. To encompass a wide range of small and large mammals in Iran, we assigned body sizes of 0.5, 10, 50, and 100 kg (Ziaie, 2008), resulting in four resistance maps for each body size scenario. Furthermore, we calculated the minimum, maximum, and mean resistance values across all cells of the resistance maps created for each body size (ranging from 1 to 100 kg). This analysis aimed to plot and understand the relationship between body size and resistance across Iran in kilocalories. It's important to note that these resistance maps provide a general overview of Iran's resistance to mammals with different body sizes, and they were not utilized for further modeling purposes.

### 2.4 Least‐cost analysis

To identify the least‐cost corridors between core habitat areas, we utilized the Linkage Mapper Toolkit (McRae & Kavanagh, 2011). Initially, Linkage Mapper creates a map known as cost‐weight distances, which involves multiplying the cell size by the value of its resistance, thereby indicating the cumulative movement cost. This map illustrates the least cumulative cost necessary to traverse between a cell and a resource. Linkage Mapper then utilizes this map to ascertain the least‐cost corridors between core habitats. Given the four body size scenarios, we adjusted the maximum dispersal distance accordingly. For small mammals with a body size of 0.5 kg, we considered a maximum dispersal distance of 50 km. For larger body sizes of 10, 50, and 100 kg, we adjusted the maximum dispersal distances to 100, 200, and 300 km, respectively. Other studies have also utilized similar thresholds for maximum dispersal distances, corroborating our approach. For example, Rahimi and Dong (2023) considered 200 km and Khosravi et al. (2018) used a maximum dispersion ability of 300 km for large mammals. Ashrafzadeh et al. (2020) set thresholds at 200 km for leopards and cheetahs, 150 km for lynxes, 100 km for caracals and palace cats, and 100 km for feral cats. Other studies have considered distances of 148 km for lynxes (Samelius et al., 2012), 217 km for cheetahs, and 82 km for leopards (Farhadinia et al., 2018).

To determine whether small and large mammals share the same least‐cost paths and to identify any differences between these paths, we extracted the geographic locations of least‐cost paths (LCPs) passing over DEM cells for four groups of mammals under study. However, we limited our analysis to corridors of mammals with a weight of 500 g because, based on their dispersal characteristics considered in this study, they are expected to have fewer corridors naturally. Nonetheless, we aimed to investigate if these corridors are the same as those identified for other mammal groups.

### 2.5 Barrier analysis

Barriers, that hinder the movement of large mammals between their core habitat areas, have received insufficient attention in past research (McRae & Kavanagh, 2011). In the context of corridor analysis, Linkage Mapper is capable of identifying barriers that obstruct the establishment of corridors. These barriers represent areas in the landscape that significantly impede the connectivity between two points (Mariela et al., 2020; McRae et al., 2012). Linkage Mapper assesses the effect of removing each barrier on the connectivity of linkages. This evaluation is conducted through moving window analysis, where the radius of the window corresponds to the size of the barriers the user intends to detect. For our study, we set the window size to 5000–50,000 m, allowing us to effectively identify and analyze barriers within the landscape.

### 2.6 Pinch‐point mapping

In recent studies on corridor mapping, there is a notable emphasis on key concepts like pinch points, which are increasingly recognized as vital conservation targets within corridors (Liu et al., 2018; Rahimi & Dong, 2023; Sütünç, 2021). Pinch points are identified by Linkage Mapper based on the width of the corridors. The width of these raster corridors, also known as the cut‐off distance, can extend up to several kilometers. For instance, previous studies have set the cut‐off distance at 75 km for species like the American black bear and the Canadian lynx, while values of 10 km and 25 km were used for mountain goats and other species, respectively (Group, 2010). In our study, we set the width of the cut‐off distance at 10–30 km, enabling us to effectively identify pinch points within the landscape for different mammal species.

### 2.7 Land use, elevation, and roads overlapping with LCPs

**FIGURE 2:**
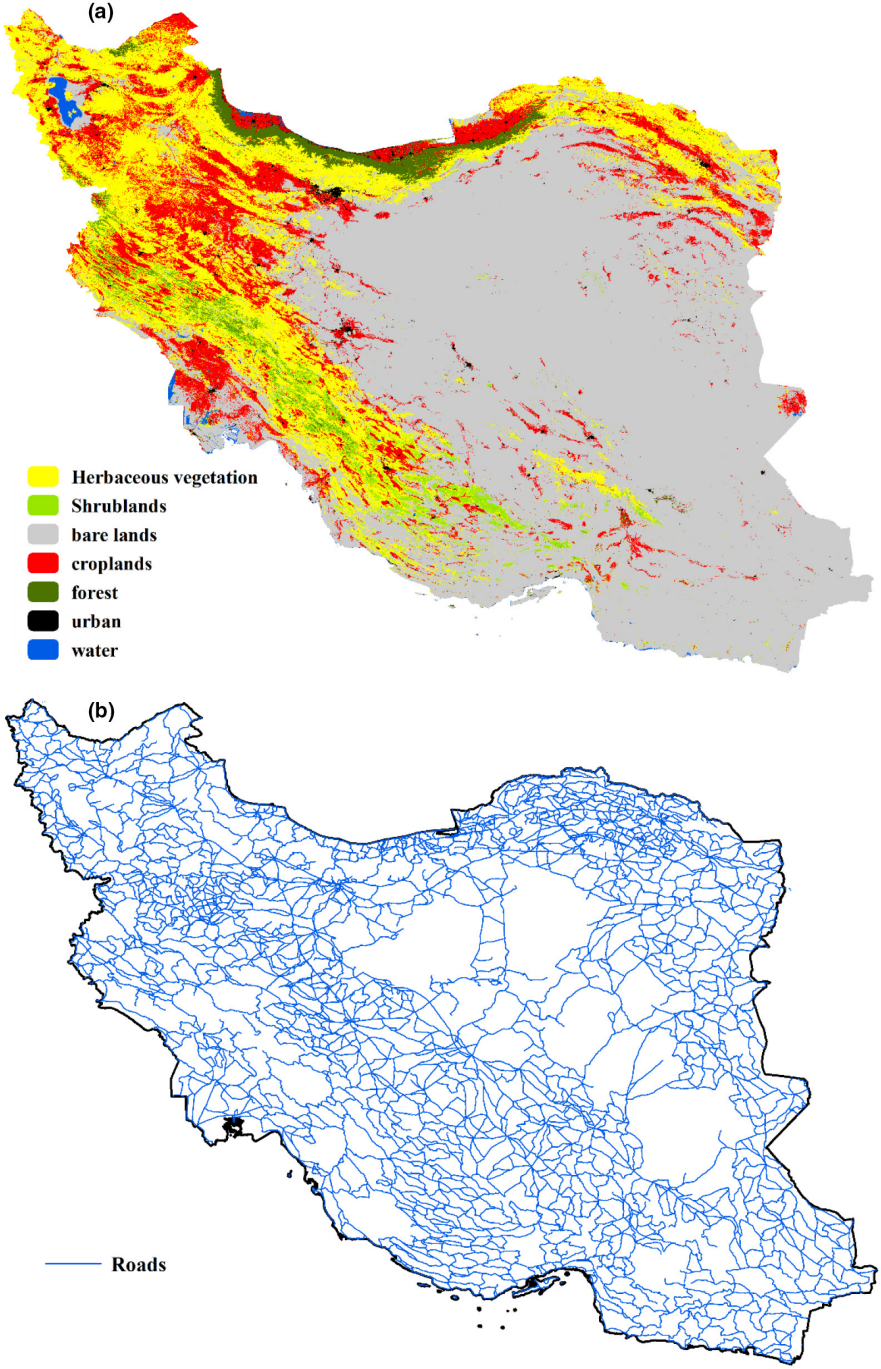
Land use (a) and road network (b) of Iran.

To understand the typical land cover through which identified corridors should pass, we overlapped the corridors for four groups of mammals with the land use map of Iran from 2019, produced by Copernicus (Mariela et al., 2020). Our focus was specifically on croplands and urban areas (Figure 2a), which can hinder mammal movement. Additionally, we calculated the average elevation of each identified corridor. Furthermore, using the ‘gIntersection’ function from the ‘rgeos’ R package (Bivand et al., 2017), we determined the locations of intersections between the corridors and the road network of Iran (Figure 2b). These intersections can be viewed as barriers to animal movement. We then can compare the results of these overlaps with the enerscape approach, which does not consider human infrastructure and land use in resistance map creation. This analysis allowed us to gain insights into the influence of different land covers and human infrastructure on the identified corridors, as well as to evaluate the effectiveness of the enerscape approach in considering these factors.

## 3 RESULTS

### 3.1 Resistance maps

**FIGURE 3:**
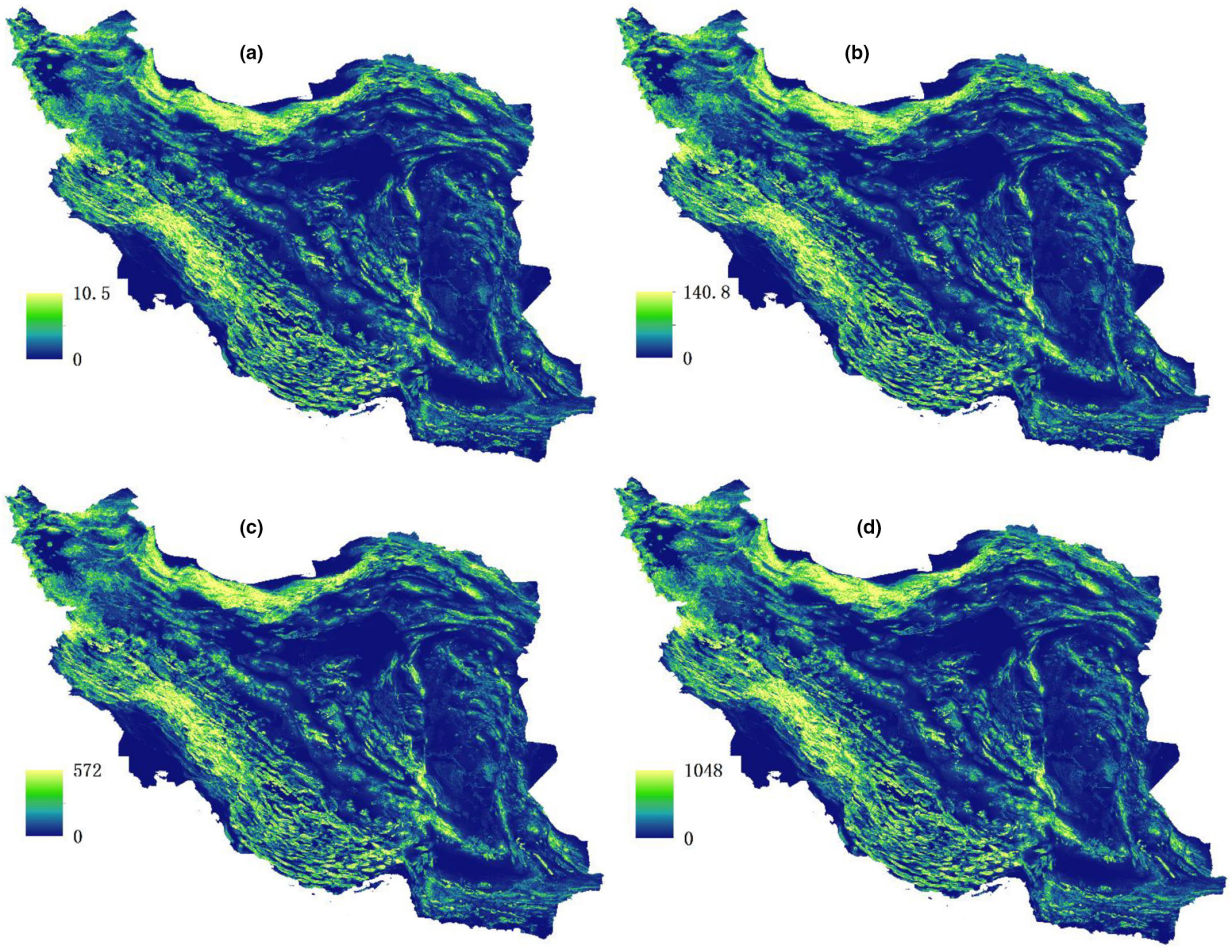
Resistance maps for different body sizes (a), 0.5 kg (b), 10 kg, (c) 50 kg, (d) 100 kg (unit = kcal).

Figure 3 depicts resistance maps showcasing the energy usage or metabolic rates for mammals of various body sizes across different regions. Lighter shades on the maps represent higher energy expenditure, measured in kilocalories (kcal). For instance, in Figure 3a, the maximum energy usage for a mammal weighing 0.5 kg is 10.5 kcal in Iran, while for a mammal weighing 100 kg, it amounts to 1048 kcal. These maps highlight how energy expenditure varies for mammals of different sizes in different parts of Iran.

### 3.2 Least‐cost paths

Table 1 provides insights into the characteristics of identified corridors for different mammal groups, highlighting variations in corridor length, elevation, intersections with roads, and passage through croplands and urban areas, reflecting the varying challenges faced by mammals of different sizes in navigating their habitats. The number of corridors identified varies across mammal groups, ranging from 206 for 500 g mammals to 447 for 100 kg mammals, reflecting the increasing dispersal ranges as mammal size increases. Mean corridor lengths also exhibit a similar trend, increasing from 39 km for 500 g mammals to 117.6 km for 100 kg mammals. Interestingly, the mean elevation of corridors follows a different pattern, with 500 g mammals exhibiting a higher mean elevation compared to 100 kg mammals. This divergence may stem from larger mammals having more corridor options and potentially traversing more low‐elevation areas. The number of intersections between corridors and roads increases with mammal size, indicating a greater likelihood of encountering barriers such as roads for larger mammals. Furthermore, corridors passing through croplands show a similar trend, with larger mammals having to negotiate more extensive croplands, potentially hindering their movement. Urban areas also present barriers to mammal movement, with smaller mammals facing more obstacles due to their limited alternative paths compared to larger mammals. In addition, there is a notable overlap in least‐cost paths between 50 and 100 kg mammals, suggesting similar corridor patterns for these larger mammal groups.

**TABLE 1:** Least‐cost paths topographic and land use characteristics.

	Number	Mean (km)	Mean elevation	Roads intersection	Cropland (km)	Urban (km)
500 g	206	39 (35)	1276 (643)	579	9.3 (13)	2.1 (3)
10 kg	333	72.7 (59)	1309 (609)	1767	13.7 (19)	2.1 (3)
50 kg	434	110.7 (97)	1172 (612)	3115	14.3 (19)	1.9 (3)
100 kg	447	117.6 (104)	1150 (612)	3255	14.3 (19)	1.9 (3)

**FIGURE 4:**
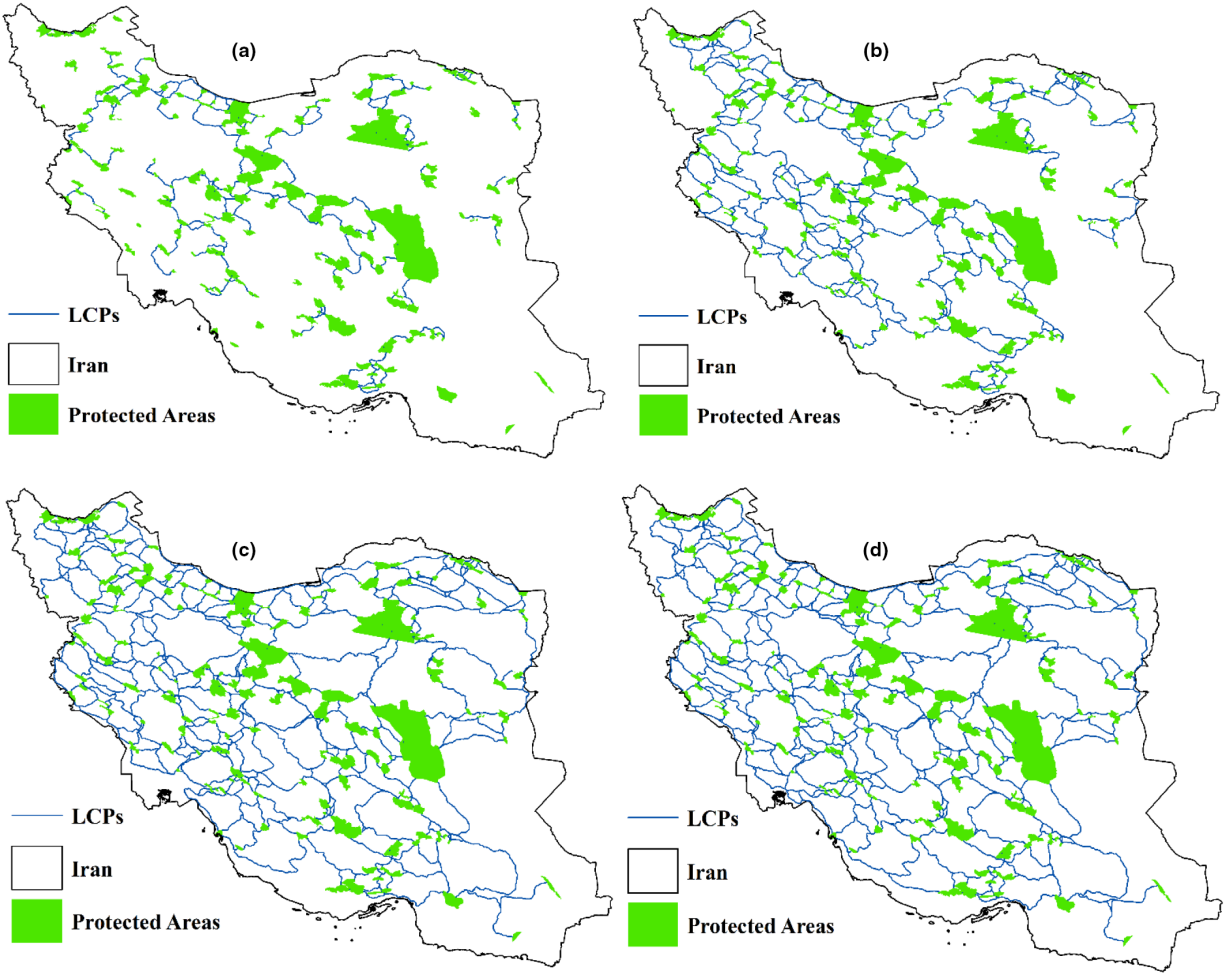
Least‐cost paths for different scenarios (a), 0.5 kg, maximum dispersal 50 km (b), 10 kg, maximum dispersal 100 km (c) 50 kg, maximum dispersal 200 km (d) 100 kg, maximum dispersal 300 km.

Figure 4 portrays least‐cost pathways for various scenarios of mammal dispersal, each defined by specific mammal weights and maximum dispersal distances. The fig suggests that as mammals' dispersal ability increases, the network of least‐cost pathways expands, introducing new routes between protected areas. This implies that in the event of disruption along one pathway, mammals have alternative corridors available, as multiple pathways connect the protected areas.

### 3.3 Overlap between LCPs

In the previous section, we noted that mammals weighing 500 g had 206 least‐cost paths identified by Linkage Mapper based on a maximum dispersal distance of 50 km. Our investigation in ArcGIS software revealed that these 206 corridors pass over 10,249 cells. We then extracted the latitude and longitude of these cells and compared their overlap with the extracted locations for the other three mammal groups. Our analysis showed a 100% overlap between cells identified for drawing least‐cost paths for all groups of mammals. However, presenting these results in a table would be redundant, as it would simply show a single value of 100% overlap for corridors. Instead, we decided to visually illustrate a small subset of Iran's corridors between protected areas. This visualization demonstrates how, based on the enerscape approach, the lowest elevation receives the minimum resistance, leading to the least‐cost paths passing over these cells.

**FIGURE 5:**
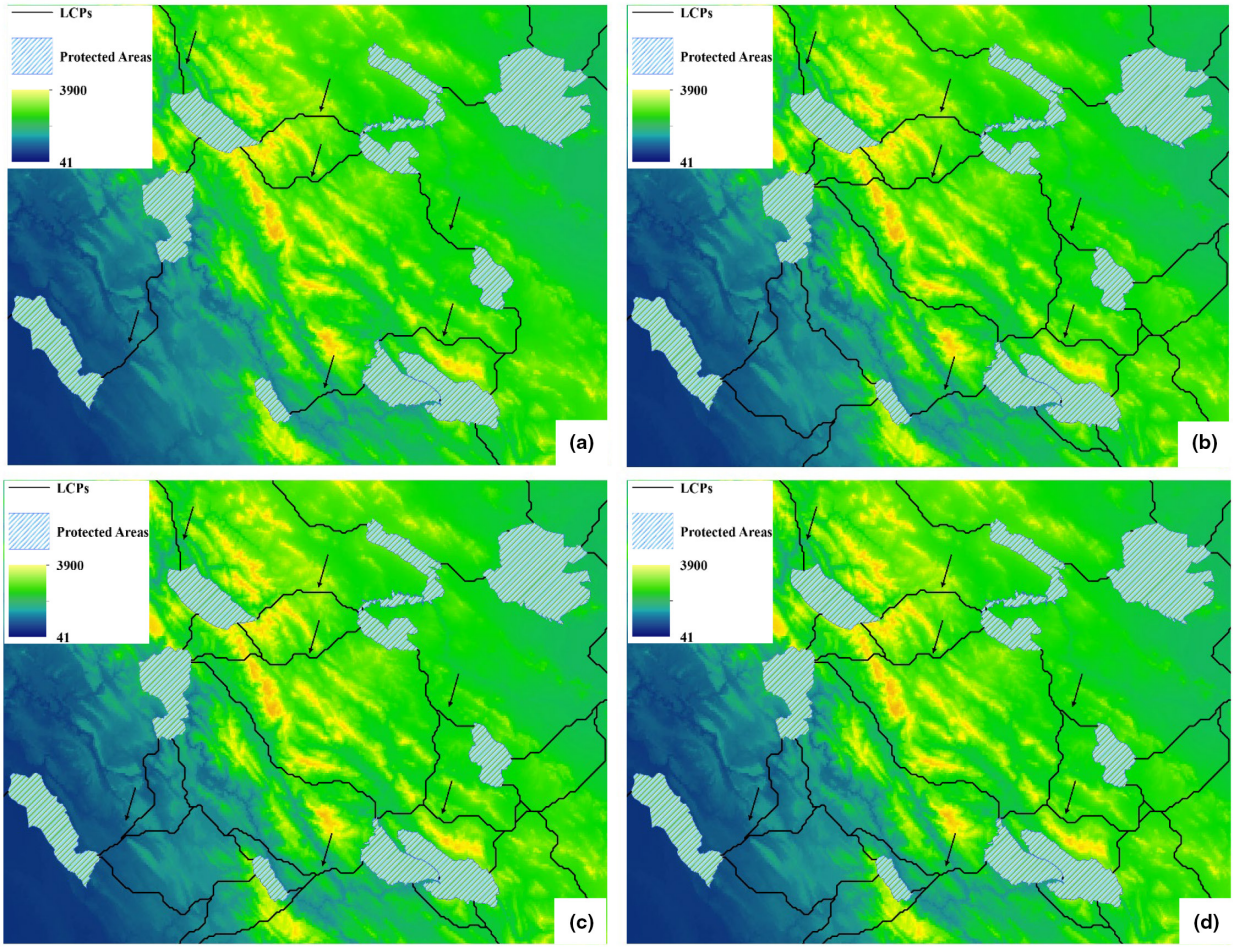
Overlap between least‐cost paths of mammals (a) 500 g, (b) 10 kg, (c) 50 kg, and (d) 100 kg on DEM.

Figure 5 illustrates the overlap of least‐cost paths for mammals of different weights: (a) 500 g, (b) 10 kg, (c) 50 kg, and (d) 100 kg. As depicted in the figure, panel a shows the least‐cost paths for 500 g mammals, while panels b, c, and d represent increasing dispersal ranges, resulting in a higher number of corridors for larger mammals. However, we have highlighted certain corridors with arrows to emphasize that, upon comparison, some corridors for 500 g mammals are also identified as least‐cost paths for larger mammal groups. For instance, between areas 1 and 2, there are no paths for 500 g mammals, but as dispersal ranges increase, overlapping corridors emerge, particularly for mammals weighing 50 and 100 kg, where the same path network is observed.

**FIGURE 6:**
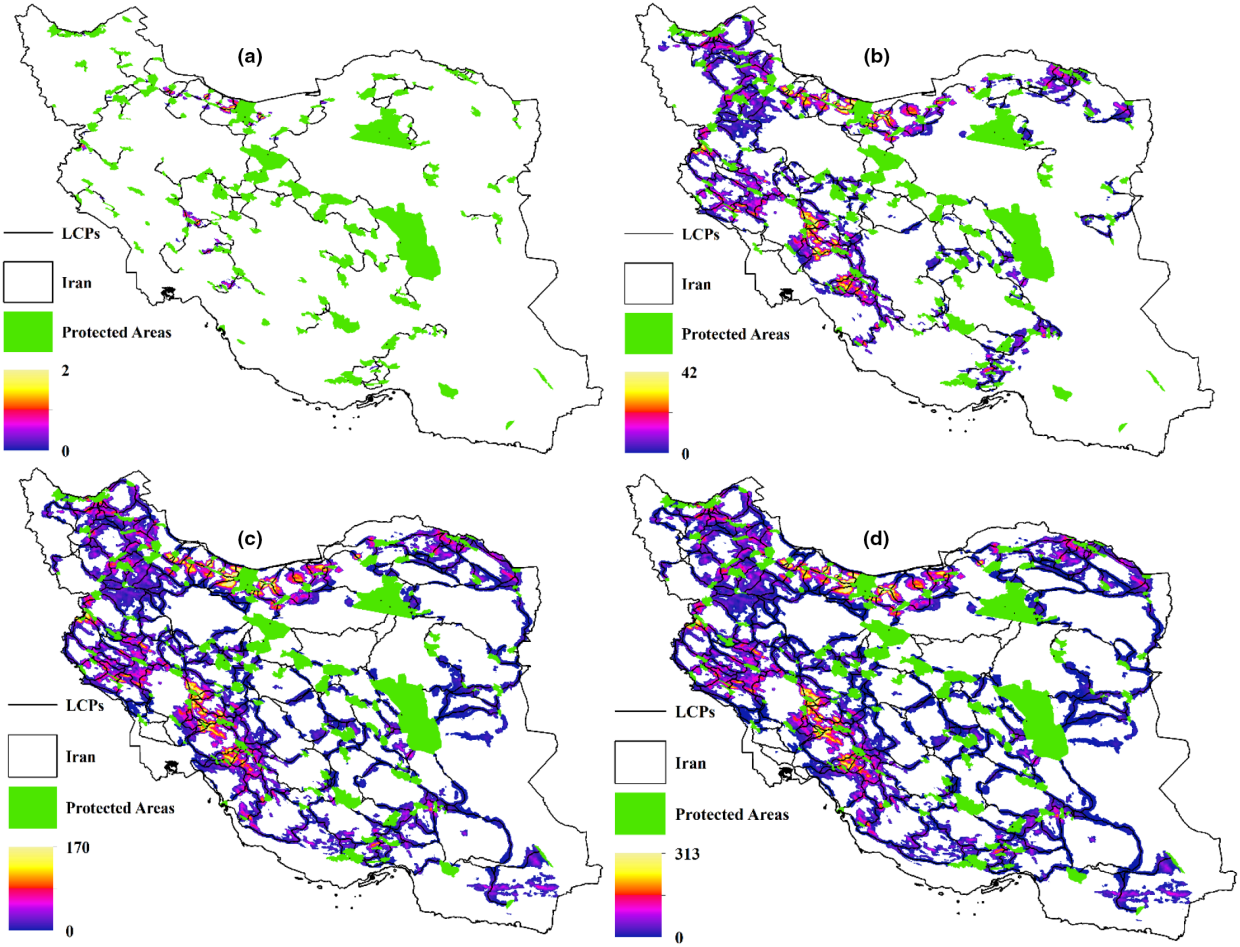
Barriers in the way of Least‐cost paths for different scenarios (a), 0.5 kg, maximum dispersal 50 km (b), 10 kg, maximum dispersal 100 km (c) 50 kg, maximum dispersal 200 km (d) 100 kg, maximum dispersal 300 km (unit = kcal).

**FIGURE 7:**
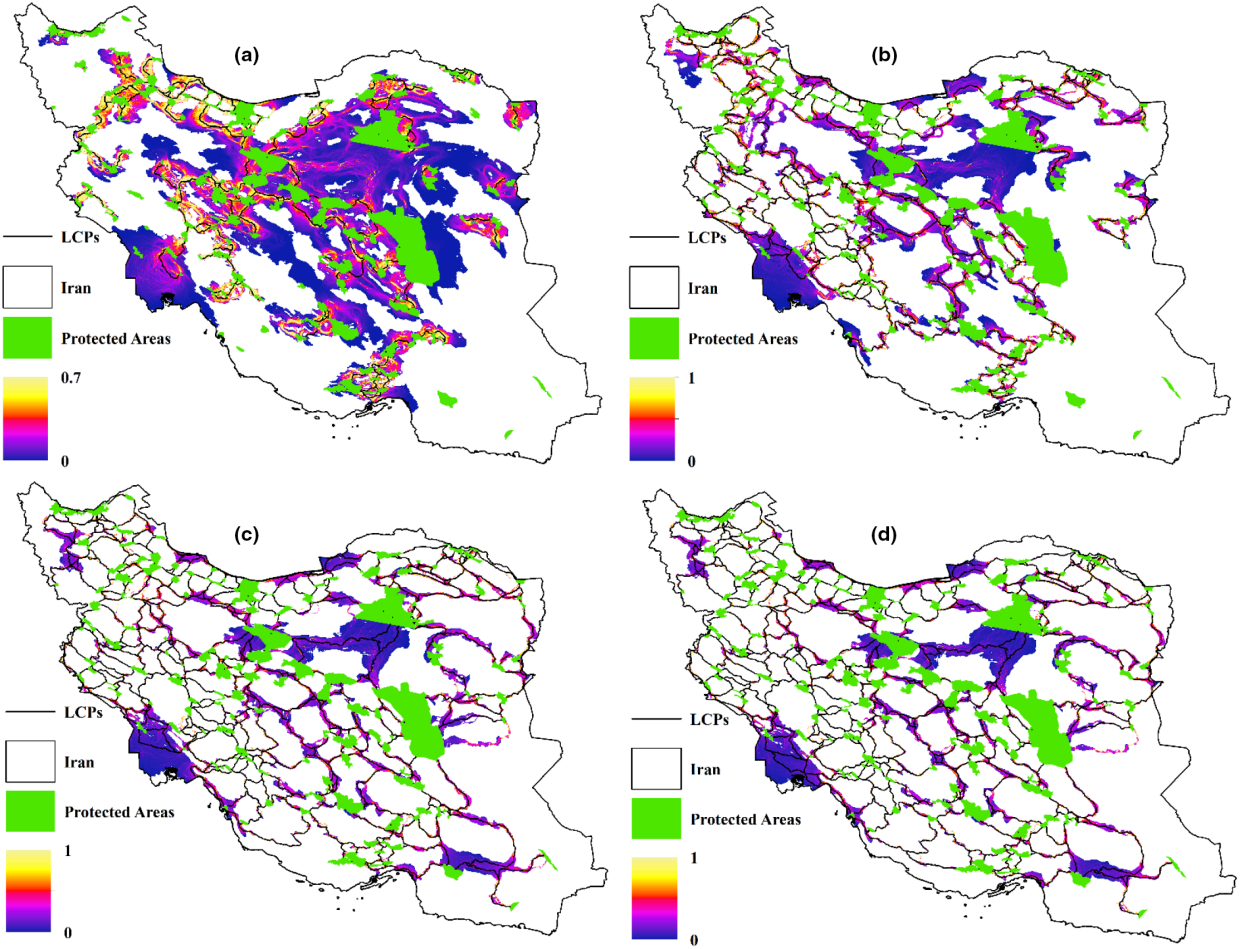
Pinch points of least‐cost paths for different scenarios (a), 0.5 kg, maximum dispersal 50 km (b), 10 kg, maximum dispersal 100 km (c) 50 kg, maximum dispersal 200 km (d) 100 kg, maximum dispersal 300 km.

Figure 6 illustrates barriers obstructing the least‐cost paths across various scenarios, with each scenario defined by specific mammal weights and maximum dispersal distances. The fig shows lighter colors as barriers along corridors, indicating that some corridors are barrier‐free while others have obstacles along their paths. In this fig, the units are based on a resistance map created using kcal values for each mammal group. Consequently, for larger mammals, the barrier values are larger compared to smaller ones. Figure 7 depicts pinch‐points along the least‐cost paths across various scenarios, each associated with specific mammal weights and maximum dispersal distances. Lighter colors in the fig indicate the presence of pinch points. In this fig, pinch points are scaled within a range of values from 0 to 1, where a value of 1 represents the highest conservation priority.

## 4 DISCUSSION

In this research, we aimed to identify the corridors connecting protected areas in Iran based on the energy expenditure of mammals and determine the overlap between identified corridors. We conducted analyses across four scenarios of mammal weight. We used Linkage Mapper, which identifies paths with the lowest cost based on resistance maps, and utilized DEM data to calculate energy usage and create resistance maps. Consequently, it was anticipated that the potential least‐cost paths identified would traverse DEM cells with the lowest elevation for each studied mammal group. For instance, 206 corridors were identified for mammals weighing 500 g, and these same corridors were also identified as least‐cost paths for 100 kg mammals. However, due to the larger dispersal range of the latter group, their corridor count increased to 447. This outcome suggests that the enerscape approach for corridor identification yields the same least‐cost paths for mammals of the same size and dispersal range, without differentiating between their behavioral ecology, such as whether they are carnivores or herbivores. Consequently, one reason previous studies utilized Species Distribution Models (SDMs) to create resistance maps is that it allows for the creation of different resistance maps for each species, resulting in different identified corridors for each species. However, enerscape is a recently introduced approach, and a comprehensive study was needed to examine its strengths and weaknesses.

Since enerscape identifies the same paths as least‐cost paths, the locations of pinch‐points and barriers are also the same for each group of mammals, which is another noteworthy aspect of this approach. As this approach does not incorporate human infrastructure and land use in the energy usage calculations of animals and subsequently in the creation of the resistance map, we analyzed by overlapping the identified corridors with the land use map and road network of Iran. We discovered that for mammals weighing 100 kg, there were 3255 intersections with roads, each of which can act as a barrier to their movement. However, since we used DEM for the resistance map, barriers were also identified based on elevation, likely representing locations with the highest elevation along the corridors. Additionally, our analysis revealed that mammals must traverse urban areas and several kilometers of croplands, each of which can hinder their movement.

Therefore, it appears necessary to include human infrastructures in the resistance map created by this approach to obtain more realistic paths and barriers between protected areas. Also, Santini et al. (2013) conducted an exhaustive meta‐analysis encompassing 327 studies investigating the dispersal patterns of 164 mammal species. Utilizing both linear and non‐linear regression analyses, they examined the associations between body size, home range area, and dispersal distance. Their findings revealed that the linear relationships between home range and body size with dispersal distance were influenced by a multitude of life history traits and ecological factors. Hence, considering dispersal considerations can significantly impact the identified corridors, suggesting that incorporating this aspect would enhance corridor identification efforts.

Our analysis further revealed that regions such as the north, south, southwest, and central parts of Iran, characterized by low altitude and topography and predominantly covered by bare lands according to the provided land use map, entail lower energy costs for mammalian movement. Expanding the dispersal ability from 50 to 300 km unveils multiple potential corridors between protected areas, providing mammals with alternative routes that vary in their energy requirements. This highlights the importance of considering the diverse topographical and land use characteristics of different regions when assessing landscape connectivity and planning conservation strategies. However, we recognize that other factors such as human presence and infrastructure, known as the ‘landscape of fear’ (Gallagher et al., 2017), particularly roads and urban areas, can significantly influence mammal migration routes (Ghadirian et al., 2019; Mohammadi et al., 2018; Parchizadeh et al., 2017; Rahimi & Dong, 2022, 2023). The concept of the ‘landscape of fear’ stems from the recognition that predators induce a fear of predation in their prey, exerting profound effects on prey physiology, behavior, and life history across ecosystems. This understanding underscores the significance of predators in ecological dynamics and broader ecosystem‐level implications. Encounters with predators introduce a significant threat, potentially nullifying an animal's future fitness depending on individual circumstances. The concepts of energy acquisition, utilization, and interactions within the ‘landscape of fear’ are interrelated paradigms that offer insights into spatial ecology and decision‐making in wild animals (Gallagher et al., 2017).

Numerous studies in Iran have incorporated climatic and anthropogenic variables to determine corridors (Ashrafzadeh et al., 2022; Khosravi et al., 2019; Mahmoodi et al., 2023; Mohammadi et al., 2021; Shahnaseri et al., 2019), our decision to solely consider energy consumption for corridor determination was deliberate, aiming to offer fresh insights in this domain. The corridors identified in our study represent the most likely or primal selective routes for mammals, as they align with energy conservation principles (Shepard et al., 2013), whereby mammals tend to favor routes with minimal energy expenditure. Comparing these corridors derived from the energy‐landscape approach with those derived from habitat suitability and human infrastructure presence facilitates valuable discussions and insights for future research in this field.

Furthermore, this study encourages researchers in this field to not only delineate corridors between habitats but also to proceed to the subsequent phase, which involves identifying the specific habitats along these corridors and pinpointing critical protection areas like pinch points. In this context, tools like Linkage Mapper emerge as pivotal for such endeavors, garnering increased attention lately for their efficacy in identifying habitat cores of large mammals in Iran. For instance, Rahimi and Dong (2023) aimed to identify the most efficient corridors connecting core habitat regions of two significant mammal species, the brown bear (*Ursus arctos*) and the Persian leopard (*Panthera pardus saxicolor*), within Iran, while also pinpointing barriers impeding these pathways. Additionally, they aim to recognize pinch points or bottlenecks crucial for mammalian conservation prioritization. To achieve this, they utilized the Linkage Mapper toolkit to model landscape connectivity. For brown bears, they found that the average length of the least‐cost corridors across 84 core habitat areas was 74 km, with an average of 179 barriers, primarily linear infrastructures such as roads, hindering migration to other regions. For the Persian leopard, they identified five core areas with an average corridor length of 397 km, encountering 36 barriers between them.

## 5 CONCLUSION

Despite differences in body size and energy requirements, many corridors are utilized by both small and large mammals, indicating common pathways that entail maximal energy expenditure. The discovery of shared corridors between small and large mammals highlights a notable aspect of the enerscape approach in identifying mammal corridors. This highlights the importance of adopting a holistic approach to resistance map creation that includes human‐related phenomena, thereby facilitating a more realistic identification of corridors and barriers for mammals. Incorporating species‐specific characteristics into resistance map creation allows for the identification of more realistic conservation priorities aimed at safeguarding wildlife populations and enhancing ecosystem resilience. Additionally, the identification of pinch points or bottlenecks highlights areas of heightened conservation importance where limited alternative routes are available. By prioritizing conservation efforts in these critical areas, we can mitigate the potential negative impacts of habitat fragmentation and promote landscape connectivity for wildlife populations. This proactive step is essential for maintaining biodiversity and supporting the long‐term health of ecosystems in Iran.

## AUTHOR CONTRIBUTIONS

**Ehsan Rahimi:** Conceptualization (lead); formal analysis (lead); methodology (lead); software (lead); writing – original draft (lead). **Pinliang Dong:** Investigation (equal); supervision (equal); writing – review and editing (equal). **Faraham Ahmadzadeh:** Resources (equal); supervision (equal); validation (equal); writing – original draft (equal).

## CONFLICT OF INTEREST STATEMENT

The authors declare that they have no competing interest.

## CONSENT FOR PUBLICATION

The authors confirm that they have no conflicts of interest to disclose concerning this publication.

## Data Availability

Shapefiles for protected areas and least‐cost corridors for four mammal scenarios in this study are accessible at https://github.com/ehsanrahimi666/Corridors.git.
